# Case of Insufficiency of the Mitral Valves

**Published:** 1879-12

**Authors:** 


Case of Insufficiency of the Mitral Valves: Treatment with Marked Improvement.—February 2, 1878, Dr. I. Royse brought to the University clinic, at Ann Arbor, Mr. G. H., who gave the following history: Age, twenty-nine; occupation, painter; unmarried; residence, Ann Harbor; health never very good; has not had many severe diseases—most severe was typhoid fever, some nineteen years ago. He says his habits have been quite regular, and that he has most of the time had good living. His father had rheumatism. Supposed cause of patient's present trouble was taking cold about four years ago. Soon after contracting the cold he was attacked with accute articular rheumatism, from which he did not obtain comple e relief for nearly a year. Some six or eight months since he began to be "short-winded" on exercise. He noticed that he began to bloat soon after this and palpitation of the heart manifested itself, accompanied by the other vague, uneasy sensations of the præcordia that characterize cases of valvular disease of the heart.

The patient presented the following objective conditions: Man in good flesh, but badly out of breath from climbing the stairs He is somewhat anæmic; feet and limbs badly swollen—in fact, so much so that he sat far forward on his chair, as if in this way to partially prevent flexing his swollen knees and hips. Abdomen very prominent, and the whole cardiac region perturbed by the violent action of the heart. Palpitation showed the apex beat to be at least an inch to the left of the nipple—(the number of pulsations per minute was not recorded, but I think that I am safe in saying that they were something over 100.) Ausculation revealed a *loud*, blowing sound at the apex, occupying the portion of the heart-sound that should have been occupied by the first sound. At, or near, the apex no first sound could be heard, it being completely obscured by the adventitious one. The blowing sound was diffused to a considerable extent over the chest in all directions, and was perfectly distinct as far around the side as the posterior boundary of the axillary region. At the base of the heart the first sound could, by careful attention, be distinguished. The second sound seemed to be natural. In both infra scapula regions moist crackling rales were heard on inspiration, indicating pulmonary œdema. Percussion and palpation at lower part of abdomen revealed ascites.

*Diagnosis.*—Insufficiency of the mitral valves; due to an old endocarditis, it, in turn, the result of rheumatism.

*Prognosis.*—Very grave.

The patient was unable to stay in the hospital all the time, but came there each afternoon for nearly two weeks, to allow the members of the class to listen to the abnormal sounds. The writer drove to his stopping place for him on several occasions, and commonly found him entirely unable to put on his shoes, and on any unusual jolt of the cutter, while on the road, the patient would cry out and gasp for breath in such a way as to cause serious apprehension of immediate dissolution. He certainly could not have been much worse and still have survived. In the meantime he had been put upon the following treatment:

℞—Elixir phosphate of iron, quinia and strychnia ............................ ℥ jv

Sig. Teaspoonful after each meal.

℞—Tr. digitalis ............................ ℥ij

Sig. Begin with five drop doses three times daily and gradually increase the dose until its physiological effect is manifest.

In addition to the above, he has given an occasional hydrogogue cathartic, and directed to use, as a drink, a solution of cream of tartar. In a short time the patient was taking ten drops of the digitals, with marked decrease of the number of heartbeats per minute.

I regret that the records do not give dates indicating the progressive movement. The patient was seen occasionally during the months of February, March and April, but no change was made in the line of treatment It was several weeks before any improvement in the bloating could be seen—about the first evidence of its decrease being his ability to get on his shoes. When the weather became warm, he was able to get out upon the street and do odd jobs of work. About July 1st the anasarca had so far diminished that he began work for a butcher, running a hand delivery cart all over the city. About this time the Professor made an examination and found the patient apparently well, except the enlargement of the heart and blowing sound at the apex. Both these abnormal conditions had been improved, but were by no means removed. The dropsy, if any existed, was not perceptible Sometime in the fall following the patient went to Jackson and began work in a railroad car shop. At one time he worked with shovel and wheel barrow, but, as he experienced trouble with his heart, he gave up such heavy employment. A letter from him, dated May 15 h, 1879, says he does any work he can find to do; can get around as lively as ever; has no swelling of the feet, and goes up and down stairs and ladders without any inconvenience. A card from Dr. N. H. Williams, of Jackson, of the same date, says the condition of the heart, as determined by a careful examination, is about the same as when he left Ann Arbor. Another card from Dr. Williams, dated September 11th, 1879, says the patient is at work in the country, and that during the summer he has "experienced little or no trouble from his once formidable heart trouble."

What the ultimate result of the case will be, is, of course, problematic, and can only be determined by time. The case is of interest for the following reasons: (1) The certainty of diagnosis; (2) The marked improvement under treatment. Of the accuracy of the diagnosis I think there can be no doubt, when, in connection with the physcal signs, we remember the symptoms and history. Considering the degree of dyspnœa, the amount of dropsy, and the marked œdema of the lungs, all undoubtedly due to the regurgitation, the improvement thus far has been, at least, as satisfactory as could be asked. Should further developments of interest in the case come to my knowledge, I will report them through *The Physician and Surgeon*. I shall do so whether such developments be favorable or unfavorable, for I believe the progress of the case thus far makes it an interesting one.—*The Physician and Surgeon.*

